# Acute Myeloid Leukemia: Diagnosis and Evaluation by Flow Cytometry

**DOI:** 10.3390/cancers16223855

**Published:** 2024-11-17

**Authors:** Feras Ally, Xueyan Chen

**Affiliations:** 1 Department of Laboratory Medicine and Pathology, University of Washington, Seattle, WA 98195, USA; fally@uw.edu; 2 Translational Science and Therapeutics Division, Fred Hutchinson Cancer Center, Seattle, WA 98109, USA

**Keywords:** acute myeloid leukemia, flow cytometry, immunophenotype, genetic aberrations, machine learning

## Abstract

With recent technological advances and significant progress in understanding the pathogenesis of acute myeloid leukemia (AML), the updated fifth edition WHO Classification (WHO-HAEM5) and the newly introduced International Consensus Classification (ICC), as well as the European LeukemiaNet (ELN) recommendations in 2022, require the integration of immunophenotypic, cytogenetic, and molecular data, alongside clinical and morphologic findings, for accurate diagnosis, prognostication, and guiding therapeutic strategies in AML. Flow cytometry offers rapid and sensitive immunophenotyping through a multiparametric approach and is a pivotal laboratory tool for the classification of AML, identification of therapeutic targets, and monitoring of measurable residual disease (MRD) post therapy. The association of immunophenotypic features and recurrent genetic abnormalities has been recognized and applied in informing further diagnostic evaluation and immediate therapeutic decision-making. Recently, the evolving role of machine learning models in assisting flow cytometric data analysis for the automated diagnosis and prediction of underlying genetic alterations has been illustrated.

## Simple Summary

Acute myeloid leukemia (AML) is a heterogeneous group of diseases characterized by the clonal proliferation of immature myeloid precursors. The diagnosis and classification involve a comprehensive approach to integrate clinical, morphologic, immunophenotypic, and genetic criteria. Flow cytometry plays an indispensable role in evaluating AML. The correlation between immunophenotypic profiles and specific genetic aberrations in AML is increasingly being recognized, helping to guide more detailed diagnostic tests and immediate treatment choices. Recent advancements include the use of machine learning techniques to enhance the flow cytometry data analysis. These technological advances are now enabling automated diagnoses and the prediction of genetic mutations.

## 1. Introduction

Acute myeloid leukemia (AML) comprises a diverse group of hematopoietic malignancies characterized by the clonal proliferation of immature myeloid precursors (blasts and blast equivalents) [1]. AML has an annual incidence of approximately 4.2 cases per 100,000 people in the United States, with a median age at diagnosis of 69 years and a 5-year relative survival rate of 31.9% [2]. The etiology of AML is heterogeneous, with several recognized risk factors associated with disease development [3,4], including exposure to ionization radiation and/or chemotherapy (particularly alkylating agents and topoisomerase II inhibitors) leading to therapy-related AML, environmental/occupational exposures (e.g., benzene), genetic predispositions (such as inherited bone marrow failure syndromes, Down syndrome, germline mutations in *DDX41*, *CEBPA*, and *RUNX1*), antecedent myelodysplastic neoplasms (MDSs) or myeloproliferative neoplasms (MPNs), presence of clonal hematopoiesis of indeterminate potential (CHIP) mutations in *IDH1*, *IDH2*, *TP53*, *DNMT3A*, *TET2*, and spliceosome genes [5], and lifestyle factors. The pathogenesis of AML involves serial acquisition of somatic gene mutations driving leukemogenesis and disease behavior, resulting in clonal expansion of myeloid precursors and blockage of differentiation [6,7].

Diagnosing and classifying AML necessitate an integrated approach combining clinical, morphologic, immunophenotypic, and genetic data [1,8,9]. Multiparametric flow cytometry is widely available across laboratories and has become indispensable in the evaluation of hematologic conditions. In the diagnosis of AML, flow cytometry is essential for identifying immunophenotypic aberrancies of leukemic blasts and determining lineage. Emerging evidence has demonstrated a strong correlation of immunophenotypic features in a subset of AML with recurrent gene fusions [10,11] and gene mutations [12,13,14,15,16,17], facilitating prompt subclassification and therapeutic decision-making. In the evolving landscape of AML diagnostics, flow cytometric identification of surface and cytoplasmic markers provides potential targets for immunotherapy that can contribute to personalized patient care. Advances in integrating machine learning (ML) algorithms to analyze high-dimensional data have shown promising clinical application in the rapid, accurate classification and correlation of phenotypic data with genetic features and clinical outcomes [18,19,20,21,22].

## 2. Updates on the Diagnostic and Classification Scheme for AML

The classification paradigm for acute myeloid leukemia (AML) has significantly evolved over time. Since the unification of the hematopoietic and lymphoid tumor classifications under the World Health Organization (WHO) Classification systems in 2001, there have been continuous updates and refinements. These modifications and revisions have increasingly incorporated recurrent genetic abnormalities into the diagnostic criteria, as seen in the 2001 (third edition), 2008 (fourth edition), and 2017 (revised fourth edition) WHO Classification, while placing less emphasis on morphologic and immunophenotypic characteristics [23,24,25]. Cases with recurrent genetic abnormalities, including t(8;21)(q22;q22.1); *RUNX1::RUNXT1*, inv(16)(p13.1q22) or t(16;16)(p13.1;q22); *CBFB::MYH11* or t(15;17)(q24.1;q21.2); and *PML::RARA*, are categorized as AML regardless of whether the blast count meets the diagnostic criterion of 20% for AML [24,25]. This shift reflects the growing insight into the genetic aberrations driving leukemogenesis and their critical role in disease classification and prognosis.

The fifth edition WHO Classification (WHO-HAEM5) and the newly introduced International Consensus Classification (ICC) were published in 2022, both highlighting the crucial role of gene mutations in AML pathogenesis and incorporating more genetic criteria into their frameworks [8,26]. The updated classifications have expanded the list of recurrent genetic abnormalities that define specific AML subgroups, now including new variant translocations involving *RARA*, *KMT2A*, *MECOM*, and *NUP98*, as well as additional rearrangements and gene mutations such as *NPM1* and *CEBPA* (Table 1). The WHO-HAEM5 allows for the diagnosis of AML based on these genetic aberrations alone, eliminating the need for the arbitrary 20% blast count threshold. In contrast, the ICC requires a minimum of 10% blasts for an AML diagnosis in cases with recurrent genetic abnormalities, with specific exceptions. AML with *BCR::ABL1* fusion still requires at least 20% blasts under both the WHO-HAEM5 and ICC, and no evidence of chronic myeloid leukemia (CML) can be established, while AML with *CEBPA* mutations requires ≥20% blasts by the WHO-HAEM5 but only >10% by the ICC. Differentiating de novo AML with *BCR::ABL1* from the myeloid blast phase of CML can be challenging and requires a thorough evaluation of the clinical history, morphology, and cytogenetic/molecular features.

A notable addition to the ICC is the introduction of the “MDS/AML” category, which encompasses cases with 10–19% blasts lacking defining genetic abnormalities, effectively replacing the category of MDS with increased blasts (MDS-IB2) in the WHO-HAEM5. This change is supported by evidence suggesting similar clinical outcomes of patients with AML featuring ≥20% blasts and those with 10–19% blasts [27,28]. Consequently, these categories may represent a continuum within the same disease spectrum, differing only in the blast counts. The MDS/AML category is intended to streamline patient enrollment in either MDS or AML clinical trials. However, further studies are needed to standardize the implementation of these new classification schemes in designing clinical trials and for drug development [29], and to provide a clear framework for diagnosis and treatment strategies.

## 3. Flow Cytometry in the Diagnosis and Classification of AML

Flow cytometry is a powerful tool for the diagnosis of AML and post-therapy monitoring. The WHO-HAEM5 and ICC include immunophenotypic characteristics in the diagnostic criteria of AML, along with cytogenetic and molecular data [8,26]. Flow cytometry is essential for identifying and enumerating leukemic blasts, assigning lineage, and detecting aberrant immunophenotypic features, which are crucial for the diagnosis and subclassification of acute leukemia and monitoring the measurable residual disease (MRD) post treatment. The major advantages of flow cytometry include the broad instrument and assay availability, general applicability, cost-effectiveness, and rapid turnaround time for prompt therapeutic decision-making. Flow cytometry is also valuable in evaluating the surface marker expression on leukemic blasts for potential targeted immunotherapy. The main challenges are the variability across laboratories in terms of instruments, antibody panels, data analysis, and reporting, making standardization and data comparison difficult. Additionally, data interpretation requires expert knowledge, adding an element of subjectivity. Therefore, the establishment of a standardized protocol is critical for achieving interlaboratory consistency and comparability across laboratories and treatment regimens. Recent advancements have integrated machine learning algorithms into flow cytometry, enhancing the precision and efficiency of data analysis and enabling more accurate diagnosis and classification of AML.

### 3.1. Antibody Panel for Immunophenotyping

**Figure 1:**
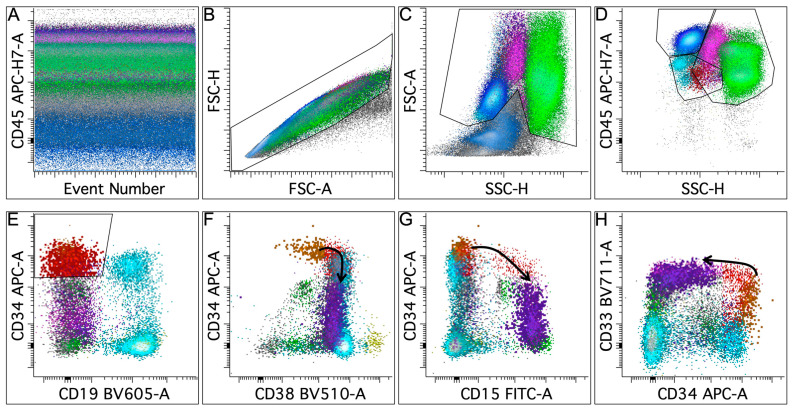
Gating strategy and normal myeloid maturation in normal bone marrow. (**A**) All the events are displayed on a CD45 versus event number plot to ensure stable data acquisition. (**B**) A singlet gate is applied to exclude doublets. (**C**) A viability gate is applied to exclude poorly viable cells and debris. (**D**) The CD45 versus side scatter (SSC) plot defines the blast gate (low CD45 and low SSC), lymphocyte gate (high CD45 and low SSC), and myelomonocytic gate (maturing myeloid cells colored green and monocytes in pink). (**E**) Events in the blast gate are displayed. A CD34-positive myeloid blast gate is generated to separate myeloid blasts from CD19-positive hematogones (colored aqua), as well as myeloid and monocytic cells. (**F**–**H**) Normal myeloid maturation from early progenitors to promyelocytes. The black line follows changes in the intensity of antigen expression. Early progenitors (highlighted in orange) express bright CD34, dim CD38, and dim CD33 without CD15. During differentiation, the progenitors gradually lose CD34 while acquiring CD15 and express higher levels of CD33 and CD38 as they reach the promyelocyte stage (highlighted in purple).

Flow cytometry facilitates the immunophenotypic evaluation of normal myeloid maturation, which is characterized by relatively conserved and reproducible antigen expression patterns. Leukemic blasts exhibit altered patterns of antigen expression that deviate from the norm, allowing them to be distinguished from normal progenitors [30,31,32]. An optimal antibody panel would enable the clear segregation of various hematopoietic populations, assessment of both the immature progenitors and myelomonocytic cells at different maturation stages, and recognition of antigenic aberrancies of leukemic blasts [33,34]. Currently, antibody panels for diagnosing AML vary between laboratories, and efforts are ongoing to standardize the reagents and methodologies to promote consistent implementation in clinical practice. The updated 2022 European LeukemiaNet (ELN) recommendations for the diagnosis and management of adult AML emphasize an integrated approach to establishing the diagnosis, with immunophenotyping by multiparametric flow cytometry required for accurate AML diagnosis [1]. The recommended panels comprise markers for precursor, myeloid, monocytic, megakaryocytic, and erythroid populations, as well as lineage markers for myeloid, T-cell, and B-cell lineages (Table 2). Standardization of these panels will ensure consistent and reliable diagnosis across different clinical settings. Gating strategies (outlined in Figure 1A–E) and methods for identifying different populations and defining immunophenotypic abnormalities are well established based on validated practices [31].

### 3.2. Antigenic Patterns During Normal Hematopoiesis

Normal hematopoiesis starts with a hematopoietic stem cell in the bone marrow, defined by high levels of CD34, low to absent levels of CD38 and CD133, a slightly higher level of CD45, and lower levels of CD13, CD33, CD117, CD123, and HLA-DR compared to CD34-positive committed myeloid progenitors. These stem cells lack antigens of lineage commitment, such as myelomonocytic antigens (CD11b, CD14, CD15, CD16, or myeloperoxidase), early B-cell antigens (CD10 or CD19), or T-cell antigen (CD3) [31,35,36,37]. As differentiation progresses toward promyelocytes, the progenitors gradually decrease the expression of CD34, CD45, and HLA-DR, while increasing the expression of CD13, CD33, CD38, and CD117, and they start to gain CD15 and CD64 (Figure 1F–H). During further maturation, promyelocytes reduce the intensity of CD13, CD33, and CD64 as they transition to the myelocyte stage, with subsequent simultaneous re-acquisition of CD13 in conjunction with CD16, both reaching high levels along with CD15 in neutrophils [30,31,38,39].

The immature monocytes (monoblasts and promonocytes) express high levels of HLA-DR and CD33, intermediate CD15 and CD64, low to absent CD13, and typically lack CD34 and CD117 [30,31,32,38,39]. With maturation, monocytes express higher levels of CD13, in parallel with CD14, CD45, CD300e, and CD312 [40,41,42].

Early erythroid progenitors exhibit increased expression of CD38, CD71, CD105, and CD117, with the slightly later acquisition of CD36, while the intensity of CD34, CD45, and HLA-DR decreases, and CD13 and CD33 are lost [31,38,39,43,44,45,46,47]. As these progenitors progress to reticulocytes, the CD71 expression remains at a high level, while the levels of CD38, CD45, CD105, and CD117 decrease, eventually leading to the loss of CD117 and CD105. Both CD71 and CD36 are lost at the mature anucleated red blood cell stage, with glycophorin A (CD235a) being retained [48]. The discrete maturation stages of megakaryocytic lineage are not well established by flow cytometry. Early megakaryocytic progenitors co-expressing CD41 (glycoprotein IIb) and CD61 (glycoprotein IIIa), and CD42 (glycoprotein 1b) are acquired with maturation [49,50,51].

### 3.3. AML with Recurrent Genetic Abnormalities

Unique immunophenotypic features have been identified in a subset of AML, which strongly correlate with both morphology and underlying genetic aberrations [10,11,52]. Recognition of these characteristic immunophenotypes is essential for prompting appropriate cytogenetic and molecular evaluation to confirm the diagnosis, guide treatment, inform prognosis, and monitor MRD.

#### 3.3.1. Acute Promyelocytic Leukemia (APL) with *PML::RARA* Fusion

**Figure 2:**
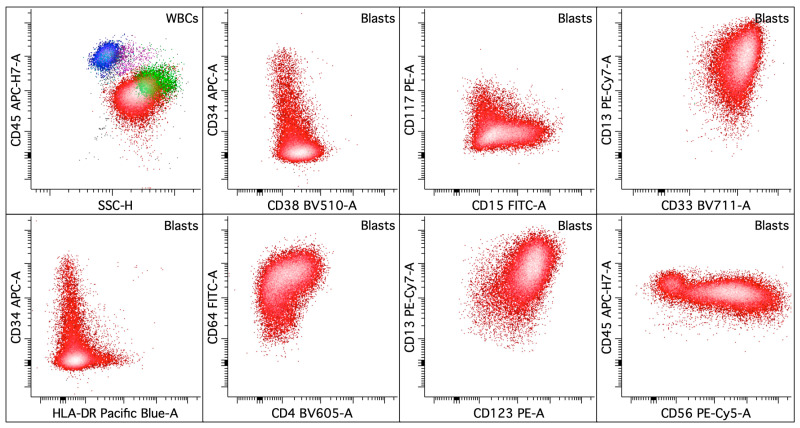
Acute promyelocytic leukemia with *PML::RARA* fusion. The upper left dot plot displays the total viable white blood cells with blasts/blast equivalents colored in red, lymphocytes in blue, monocytes in pink, and granulocytic cells in green. The rest of the dot plots selectively display leukemic blasts/blast equivalents. The leukemic blasts/blast equivalents (colored red; ~74% of the white blood cells) in the peripheral blood show variably increased SSC and express CD13 (increased, heterogeneous), CD15 (dim, variable), CD33 (uniform), CD34 (decreased to mostly absent), CD38 (dim), CD56 (major subset), CD64 (variable), CD117 (dim to absent), CD123, and HLA-DR (dim to mostly absent).

**Figure 3:**
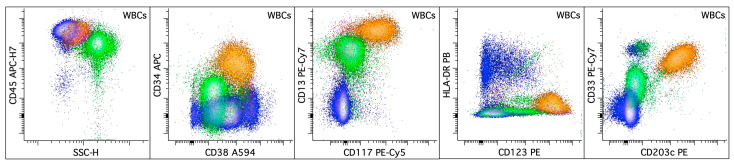
Expanded basophilic population in a patient with APL, ~7 days post all-trans retinoic acid (ATRA) treatment. All the dot plots display the total viable white blood cells with basophilic cells colored in orange, lymphocytes in blue, and granulocytic cells in green. Monocytes are few in number. The basophilic population (colored orange; ~16% of the white blood cells) in the peripheral blood expresses CD13 (bright), CD33, CD34 (dim), CD38, CD45, CD117 (dim), CD123 (bright), and CD203c without HLA-DR.

**Figure 4:**
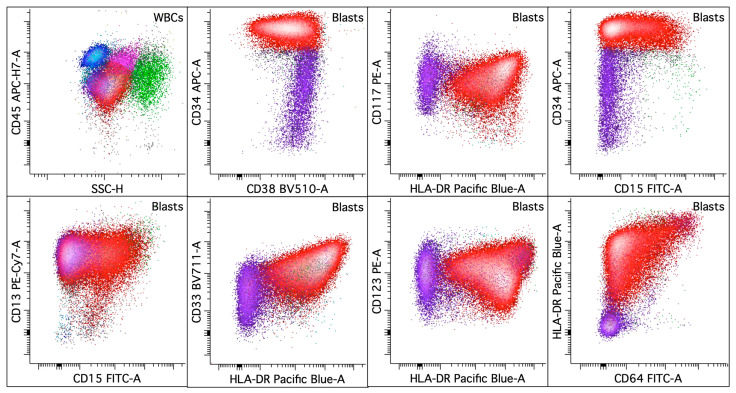
Acute myeloid leukemia with basophilic/mast cell differentiation. The upper left dot plot displays the total viable white blood cells with blasts colored in red, lymphocytes in blue, monocytes in pink, and granulocytic cells in green. The rest of the dot plots selectively display leukemic blasts. The leukemic blasts (colored red; ~55% of the white blood cells) in the bone marrow express CD13 (increased, uniform), CD15 (dim, variable), CD33 (increased), CD34 (bright), CD38 (variably decreased), CD64 (dim, variable), CD117, CD123, and HLA-DR (variable). Additionally, a CD117-positive progenitor subset (colored purple; ~7% of the white blood cells) with basophilic and/or mast cell differentiation that appears in a continuum with the first abnormal blast population is present with the expression of CD13, CD33 (dim, variable), CD34 (dim, variable), CD38, CD117, and CD123 without CD15, CD64, or HLA-DR.

APL is characterized by the clonal expansion of abnormal promyelocytes (blast equivalents) with impaired granulocytic differentiation driven by the chimeric oncoprotein, *PML::RARA*. Abnormal promyelocytes have high side scatter (SSC) and express high levels of myeloperoxidase, CD9, heterogeneous CD13, bright CD33, CD38, low to intermediate CD64, heterogeneous CD117, CD123, and low or absent CD11a, CD11b, CD18, CD34, CD133, and HLA-DR [25,53,54,55,56,57,58,59] (Figure 2). The microgranular variant of APL shows lower SSC, more frequent expression of CD2 and CD34, and higher fluorescence intensity for CD45 [53,57]. CD56 expression is detected in 15–20% of APL cases and correlates with a worse outcome [60,61,62]. Basophilic differentiation, manifested by the expression of CD203c and/or CD22, has been reported in up to one-third of APL cases at presentation [59,63,64] and is also observed in patients after all-trans retinoic acid (ATRA) and/or arsenic trioxide therapy [65,66] (Figure 3). CD203c expression is an independent predictor for severe bleeding after starting therapy and overall survival. A small subset of the blasts showing basophilic differentiation may be seen in other AML (Figure 4).

Distinguishing APL from other AML subtypes that lack CD34 and HLA-DR expression is crucial due to the life-threatening disseminated intravascular coagulation (DIC) often associated with APL, which requires prompt treatment with ATRA and arsenic trioxide to induce the differentiation of leukemic promyelocytes [67,68]. This treatment approach differs significantly from the chemotherapy used for other AML subtypes. The main differential diagnoses of APL include AML with monocytic differentiation, such as AML with *KMT2A* rearrangements, and AML with *FLT3* internal tandem duplication (ITD) and/or *NPM1* mutation. Efforts have been made to identify a specific immunophenotypic profile that can reliably differentiate APL from non-APL AML. In a study exploring 19 markers, the immune profile of low HLA-DR, low CD11a, and low CD18 was the most predictive of a diagnosis of APL [57]. By further including CD2 expression, the combination of absent HLA-DR, CD2 expression, and either absent CD11a or absent CD18 was identified in 92% of APL cases, with a specificity of 85% [58]. The presence of a distinct CD11b-negative myeloid population with the absence of CD11c has been reported to have a sensitivity and negative predictive value of 100% for APL diagnosis [55]. From an analysis using a single-tube eight-color combination, including CD11b, CD13, CD33, CD34, CD45, CD64, CD117, and HLA-DR, a unique immune profile of the absence of CD34, HLA-DR, and CD11b was found to be the most specific for the diagnosis of APL, with better specificity (93.3%) and a positive predictive value (93.1%) and similar sensitivity (90%) compared with other antigen combinations [69]. Further studies that evaluated markers as continuous variables demonstrated that the expression intensities of antigens CD13, CD56, CD64, CD117, and myeloperoxidase are significantly different between APL and non-APL AML. The combination of these five markers yielded improved diagnostic accuracy, with an area under the curve (AUC) of 0.98, sensitivity of 91.7%, and specificity of 93.1% [70]. Additionally, a combination of CD9, CD11b, CD34, CD64, CD117, and HLA-DR has been shown to definitively discriminate APL from HLA-DR-negative AML [71]. After ATRA treatment, the optimal diagnostic criteria for untreated APL lose their diagnostic sensitivity and specificity; treated APL more frequently expresses CD11b and CD11c [72].

#### 3.3.2. AML with *NPM1* Mutation and/or *FLT3 ITD*

*NPM1* mutations occur in approximately 30% of de novo AML [25]. AML with *NPM1* mutation may exhibit monocytic or myeloid differentiation, each having distinct immunophenotype [73]. Leukemic blasts in these cases typically express CD117, reduced or absent CD13, CD15, CD34, CD64, and/or HLA-DR, increased CD9, CD33, and/or CD123, and lack CD15 and CD64 [74,75]. Higher positive rates of CD4, CD9, CD10, CD11b, CD14, CD15, CD64, and HLA-DR, and lower positive rates of CD117, are detected in cases with monocytic differentiation compared with myeloid differentiation. A significant subset of AML with an *NPM1* mutation shows an “APL-like” immune profile (CD15-/CD34-/HLA-DR-/strong myeloperoxidase) [15,75,76,77,78]. Cases with an APL-like phenotype frequently display dim or absent CD13 expression (68% of cases), a higher likelihood of CD56 positivity (40% of cases), and are more likely to harbor mutations in *TET2* or *IDH1/2* and less likely in *DNMT3A.* This subtype is associated with longer relapse-free and overall survival compared to non-APL-like cases with myeloid differentiation [15]. Therefore, the combination of *NPM1* mutation and co-mutation of *TET2* or *IDH1/2* along with an APL-like immunophenotype identifies a distinct subtype of AML with a better prognosis. The expression of CD2 and/or CD34, in combination of uniform CD13 and CD64 positivity, is supportive of a microgranular variant of APL and making AML with *NPM1* mutation less likely [79].

A novel radar-plot-based flow cytometry strategy combining all the markers in each tube has been developed to distinguish APL from AML with *NPM1* mutation. The blasts/blast equivalents in APL are characterized by high expression of CD2, lack of both a monocytic component and a prominent CD11c+ population, and absence of HLA-DR and CD15. In comparison, the recurring immunophenotypic features of blasts/blast equivalents in AML with *NPM1* mutation include the presence of some admixed monocytes and a prominent CD11c population, and some expression of HLA-DR and/or CD15. These features allow radar plot analysis to confidently separate all the hypergranular APL from AML with *NPM1* mutation [12].

**Figure 5:**
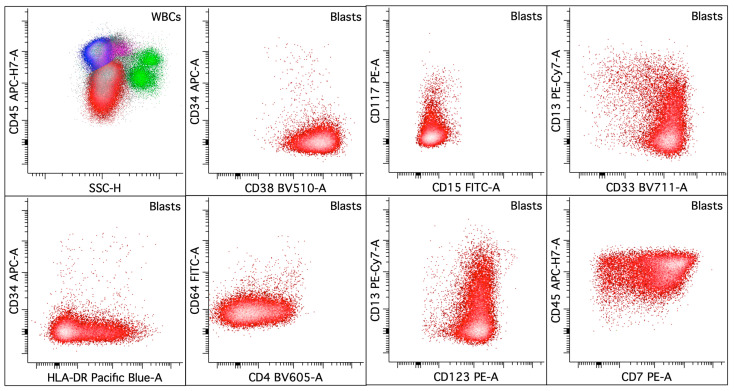
Acute myeloid leukemia with *FLT3 ITD* and *NPM1* mutations. The upper left dot plot displays the total viable white blood cells with blasts colored in red, lymphocytes in blue, monocytes in pink, and granulocytic cells in green. The rest of the dot plots selectively display leukemic blasts. The leukemic blasts (colored red; ~63% of the white blood cells) in the bone marrow express CD7, CD13 (variably decreased to absent), CD33 (uniform), CD38 (bright), CD117 (dim to mostly absent), CD123 (uniform), and HLA-DR (variably decreased to absent) without CD34 or CD64. While some immunophenotypic features overlap with acute promyelocytic leukemia (APL), SSC and the levels of CD13 of the leukemic blasts are lower than what is typically seen in APL.

For AML with *NPM1* and *FLT3 ITD* mutations (Figure 5), one study reported that an immune profile of the absence of CD34 and HLA-DR in non-APL AML is strongly associated with *NPM1* and *FLT3 ITD* co-mutations compared to the CD34+/HLA-DR− non-APL AML group [80]. The co-occurrence of *NPM1* and *FLT3 ITD* mutations was exclusively seen in the CD34-/HLA-DR− group and not seen in the CD34+/HLA-DR− group. Another study identified a leukemia-associated immunophenotype (LAIP) of CD25+/CD34+/CD99+/CD123+ that is strictly associated with *FLT3 ITD* in AML with *NPM1* mutation and a normal karyotype [81]. The proportion of CD25+/CD99+/CD123+ cells within the CD34+ population is significantly higher in the group with both *FLT3 ITD* and *NPM1* mutations compared to cases with only *FLT3 ITD* mutation. However, a recent study using the EuroFlow AML/MDS panel and principal component analysis (PCA) to characterize the LAIP in AML did not identify a specific LAIP that is associated with *FLT3* mutations. While the expression of CD123 and CLEC12A showed correlation with AML with *FLT3* mutations with or without *NPM1* mutations, the low AUC indicated the limited utility of these markers in predicting the mutational status of *FLT3*. The conclusion indicates that the utility of using immunophenotypes, CD123 and CLEC12A combined with CD34 and CD117 as surrogate markers for *FLT3* mutational status in AML is limited [14]. Large-scale studies are needed to elucidate the immunophenotypic features driven by *FLT3* mutations and the potential utility for diagnosis and MRD detection.

#### 3.3.3. AML with *KMT2A* Rearrangement

AML with *KMT2A* rearrangements typically shows monocytic or myelomonocytic differentiation [25], with the monoblasts and promonocytes (blast equivalents) having intermediate to high SSC and bright CD33 without CD34. While this phenotype is similar to that seen in APL, the characteristic features, including higher levels of CD15 and CD64, frequent HLA-DR expression, and low to absent myeloperoxidase, in the blast equivalents [25,31,82] generally differentiate AML with *KMT2A* rearrangement from APL.

A comprehensive immunophenotypic characterization of AML with *KMT2A* rearrangement revealed five distinct immunophenotypic subgroups of blast equivalents, immature monocytic, mature monocytic, APL-like, myelomonocytic, and myeloblastic [83]. There is a strong correlation between these immunophenotypic subgroups and other clinical and cytogenetic features. For example, t(11;19)(q23;p13.1) *ELL::KMT2A* was the most common rearrangement in the mature monocytic phenotype, followed by t(9;11)(p22;q23) *MLLT3::KMT2A*, whereas t(9;11) (p22;q23) *MLLT3::KMT2A* was the most frequent rearrangement in the immature monocytic, APL-like, and myelomonocytic phenotypes. Mutations in the RAS pathway were detected in >50% of cases in all the subgroups except that no mutations were found in the APL-like subgroup. AML-harboring t(6;11)(q27;q23) *AFDN::KMT2A* often has myeloblasts with a CD34+/CD117+/CD13+/CD33+/HLA-DR+ phenotype. A major immunophenotypic switch is frequently observed at disease relapse.

The blasts in AML with *CBFB* rearrangement are also characterized by myelomonocytic differentiation [25]. A recent study assessed the immunophenotypic features of AML with *CBFB* rearrangement in detail and described that the myeloblasts were positive for CD34 and CD117 in all cases and frequently positive for myeloperoxidase. The most common abnormalities included decreased CD38 and HLA-DR and increased CD13 and CD123 [84]. Monocytes were increased with a mature phenotype, expressing CD4, CD33, CD64, CD123, and HLA-DR in all the tested cases. CD56 expression on the monocytes was seen in occasional cases.

#### 3.3.4. AML with *RUNX1::RUNX1T1* Fusion

AML with *RUNX1::RUNX1T1* fusion exhibits a distinct immunophenotypic profile that has been well characterized [25,85]. The blasts typically display high levels of CD34, HLA-DR, myeloperoxidase, and CD13, with relatively weak expression of CD33. Co-expression of B-cell markers such as CD19, PAX5, and/or cytoplasmic CD79a (cCD79a) is frequently seen. CD56 is expressed in a subset of cases with *KIT* mutations and is associated with an adverse prognosis. Recognizing this immunophenotypic pattern is crucial to prevent misclassification of this AML subtype as mixed-phenotype acute leukemia (MPAL).

A flow cytometric scoring system has been generated for the rapid diagnosis and discrimination of AML with *RUNX1::RUNX1T1* fusion from other types of AML [86]. Six immunophenotypic features, including high-level expression of CD34 in blasts, aberrant expression of CD19, cCD79a, and CD56 in blasts, co-expression of CD56 in granulocytes, and maturity disturbance in granulocytes, were chosen to construct a flow cytometry score. The study demonstrated the utility of this score for the prompt diagnosis of AML with *RUNX1::RUNX1T1* fusion, with an AUC of 0.952 when the score was ≥3 points. CD19 expression in combination with *KIT* mutation has been shown to confer prognostic significance. CD19 expression on myeloblasts was the only independent factor predicting lower relapse rates, while CD19 negativity was the sole significant risk factor for relapse [87,88]. Further, *KIT* mutation and CD19 negativity correlated with the highest relapse risk.

#### 3.3.5. AML with CCAAT/Enhancer-Binding Protein Alpha (*CEBPA*) Mutation

Mutations in *CEBPA* occur in ~10% of AML. Emerging evidence supports the notion that *CEBPA* with in-frame mutations in the basic leucine zipper (bZIP) domain is strongly associated with better overall survival and a lower risk of relapse [89,90,91]. The definition of AML with biallelic mutation of *CEBPA* in the revised fourth edition of the WHO Classification has been redefined to include single mutations located in the bZIP region of *CEBPA* by the WHO-HAEM5 and replaced with AML with in-frame bZIP *CEBPA* mutations by ICC 2022 [8,9]. AML with biallelic mutation of *CEBPA* has a distinct immunophenotype [92,93]. In AML with double-mutated *CEBPA*, blasts uniformly expressed high levels of CD34, CD117, and HLA-DR, along with asynchronous expression of CD15, CD64, CD65, and myeloperoxidase, and aberrant expression of cross-lineage antigen CD7. CD56 expression is seen in ~30% of cases. In contrast, blasts in AML with a single mutation in *CEBPA* show more heterogeneous expression of these antigens. In the maturing cells, the recurrent immunophenotypic alterations associated with double-mutated *CEBPA* include low SSC, low CD65 and higher CD64 in neutrophils, and high CD64 and low CD36 in monocytic cells. Erythroid precursors show an increased proportion of CD117/CD105-positive immature precursors and antigenic aberrations manifested as low CD36 and low CD71.

Given the technical challenges and time-consuming nature of evaluating biallelic mutations of *CEBPA*, several studies have aimed to develop screening tools to predict the presence of *CEBPA* mutations. One such classifier was defined using a set of ten parameters: CD7, CD15, CD34, CD65, CD117 on blasts; SSC and CD64 on cells of the neutrophil compartment; CD14 and CD64 on the monocytic compartment; and CD117 on erythroid cells [92]. This classifier has been validated as a reliable method for distinguishing the double-mutated *CEBPA* genotype, demonstrating 100% sensitivity and specificity, as well as perfect positive and negative predictive values. Additionally, a simple flow cytometric scoring system has been created to identify patients unlikely to carry the *CEBPA* mutation. This seven-point scoring system assigns one point each for the expression of CD7, CD13, CD15, CD33, CD34, HLA-DR, and one point for the lack of CD14. A score of ≥6 is significantly correlated with the presence of double-mutated *CEBPA* (*p* < 0.05), whereas no double mutant was recognized among patients with a score <6. This system could optimize prognostic stratification by appropriately prioritizing sample evaluation [16].

#### 3.3.6. AML with Other Gene Mutations

**Figure 6:**
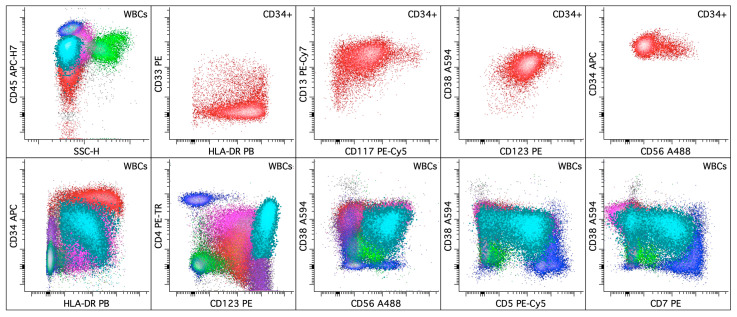
Acute myeloid leukemia with plasmacytoid dendritic cell (pDC) expansion and a *RUNX1* mutation. The upper left dot plot displays the total viable white blood cells with blasts colored in red, pDCs in aqua (highlighted), lymphocytes in blue, monocytes in pink, and granulocytic cells in green. The rest of the dot plots in the upper panel selectively display CD34+ leukemic blasts. The leukemic blasts (colored red; ~20% of the white blood cells) in the peripheral blood express CD13 (bright), CD33 (dim to mostly absent), CD34 (bright), CD38, CD56 (small subset), CD117 (decreased), CD123, and HLA-DR (variably decreased). In the lower panel, all the dot plots display the total white cells. The pDCs (highlighted in aqua; ~15% of the white blood cells) express CD4, CD5 (major subset), CD7 (major subset), CD34 (dim, variable), CD38, CD45, CD56, CD123, and HLA-DR (dim, variable) without CD2, CD13, CD14, CD33, CD64, and CD117.

Plasmacytoid dendritic cell (pDC) expansion (≥2%) has been reported in ~5% of AML [94]. In pDC-AML, *RUNX1* mutations are enriched and present in 60–70% of cases [94,95,96,97]. In pDC-AML with *RUNX1* mutations, concurrent mutations in *SRSF2*, *TET2*, *ASXL1*, and *DNMT3A* are also frequently observed [94,95]. The pDCs in pDC-AML express similar levels of CD4, CD38, CD45, CD123, CD303 (in most cases), and HLA-DR as normal pDCs, but they frequently exhibit aberrant expression of CD5, CD7, CD13, CD22, CD25, CD34, CD56, CD117, and TdT, and loss of the normal pDC markers CD2 and CD33 in a proportion of cases [94,95,96] (Figure 6). Monocytic differentiation is seen in 20–30% of cases. The myeloblasts in pDC-AML typically express CD34, CD117, HLA-DR, and uniform CD123 with variable TdT and myeloperoxidase [95]. In a subset of pDC-AML, myeloblasts showed ≥2 cross-lineage antigen expression [94]. The pDCs display an immunophenotypic profile intermediate between normal pDCs and blastic plasmacytoid dendritic cell neoplasm (BPDCN), and they demonstrate a maturational continuum, with the leukemic blasts sharing a set of antigen expression including CD13, CD34, CD117, CD123, and TdT [94,95]. The mutational profile and genetic aberrations were identical in sorted myeloblasts and pDCs [94,96]. The WHO-HAEM5 recognizes and includes clonal pDC diseases, mature pDC proliferation associated with myeloid neoplasm and BPDCN, in the category of “Histiocytic/Dendritic cell neoplasms” [8].

AML with mutated RNA-splicing factor gene *SRSF2* has a worse overall survival compared to AML with wild-type *SRSF2* [6,13]. No specific immune profile has been identified in AML with mutated *SRSF2*. The most frequently observed immunophenotypic aberrancies include decreased or absent CD33 and increased CD7, CD15, CD56, CD64, or CD123, which are similar to those seen in AML with wild-type *SRSF2*. Mutations in histone modifier genes, such as *ASXL1*, frequently co-occur with *SRSF2* mutations in AML, and their co-occurrence is associated with a particularly poor prognosis [6]. Other mutations, such as *TET2*, *STAG2*, and *IDH1/IDH2*, are more likely to be detected in co-mutated AML. 80% of AML with *SRSF2* and *ASXL1* mutations expressed two or more monocytic lineage markers (CD11c, CD14, or CD64), compared to 47% of AML cases with either *ASXL1* or *SRSF2* mutations, or with neither mutation [98]. CD2 and CD56 were more frequently expressed in co-mutated AML than non-co-mutated AML.

### 3.4. Phenotypically Defined Stages of Differentiation Arrest in AML Correlates with Genetic Drivers

A recent study established an immunophenotypic stratification of AML, showing that leukemic blasts are arrested at specific stages of myeloid differentiation [99]. The antigen expression patterns of leukemic blasts correlate with the stage of maturation arrest governed by specific genomic abnormalities, which are associated with the clinical presentation and outcomes.

AML with an immunophenotypic signature similar to that of multipotent progenitors (MPPs) is enriched in mutations in epigenetic modifiers, spliceosomes, and myeloid transcription factors, mainly *RUNX1* and *ETV6*. Multipotent hematopoietic stem cells (HSC) and MPPs had the highest levels of CD34+/CD38-/CD123+ leukemic stem cells (LSCs). Bi-allelic *CEBPA* mutations and core-binding factor (*CBF*) abnormalities are specific to the granulocyte–monocyte progenitor (GMP)-like group. AML with an immunophenotypic signature similar to monocyte progenitors (MPs) and granulocyte progenitors (GPs) is phenotypically negative for CD34, positive for myeloid markers CD13, CD33, and variable for CD117, with varying levels of HLA-DR expression (≥20% for MP-like and <20% for GP-like). *NPM1* mutations are frequently observed in both the MP- and GP-like groups, with co-mutations of *DNMT3A* and *FLT3* associated with the MP-like group and co-mutations in *TET2* with the GP-like group. *IDH1* and *IDH2* mutations, which are largely mutually exclusive with *TET2*, are also enriched in the GP-like group. *KMT2A* fusions are enriched in the MP-like group, albeit with a modest frequency.

Leukemic stem cells (LSCs), characterized by CD34+/CD38 dim/-, have emerged as a strong prognostic factor in AML [100,101,102]. Higher frequencies of LSCs at diagnosis and in the post-therapy setting are associated with adverse outcomes. An antibody panel for flow cytometric assays designed to identify LSCs has been developed and clinically validated [103,104,105]. The list of LSC-associated antigens is growing with the continuous addition of new markers, including CD9, CD25, CD45RA, CD47, CD96, TIM-3, CD123, and CD371 [104,106,107,108,109,110,111,112]. Overexpression of certain LSC antigens has been described in AML with specific genetic aberrations, for example, CD123 in AML with *NPM1* mutation [17], CD47 in AML with *CBFB::MYH11* fusion and AML with *NPM1* mutation but not *FLT3 ITD* [113], and TIM3 in AML with *RUNX1::RUNXT1* fusion, *CBFB::MYH11* fusion, and *CEBPA* mutations [114].

Integrating morphologic features and immunophenotypic classification will be greatly advantageous for predicting the underlying genetic characterizations and initiating therapeutic planning. Research on cell surface proteins (surfaceome) and proteome is emerging as a promising complementary tool to genomics, aiding in elucidating cancer pathogenesis and identifying cancer-specific surface proteins as biomarkers with diagnostic, prognostic, and therapeutic potential [115,116]. Despite their promise, the identification of clinically relevant biomarkers and disease subtypes through these methods is currently limited.

### 3.5. AML with Challenging Immunophenotypes

#### 3.5.1. Acute Erythroid Leukemia (AEL)

**Figure 7:**
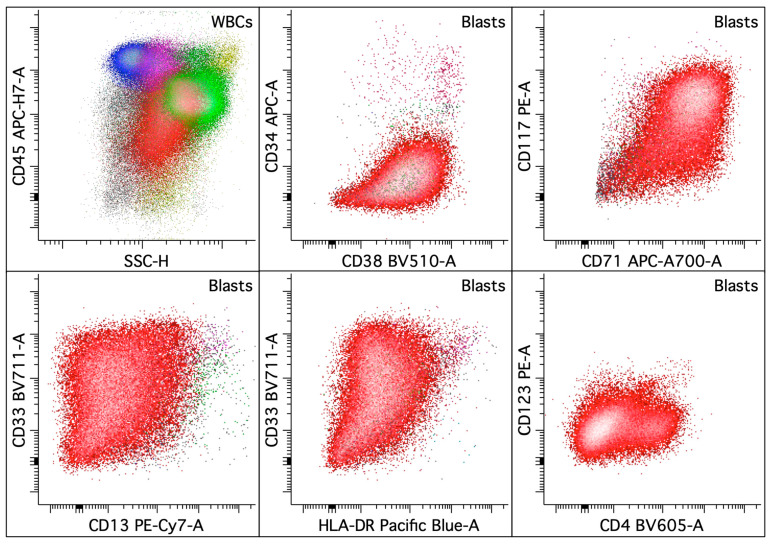
Acute myeloid leukemia with erythroid differentiation. The upper left dot plot displays the total viable white blood cells, with blasts colored in red, lymphocytes in blue, monocytes in pink, and granulocytic cells in green. The rest of the dot plots selectively display leukemic blasts. The leukemic blasts (colored red; ~22% of the white blood cells) in the bone marrow abnormally express CD4 (dim on a small subset), CD13 (dim, variable), CD33 (variable), CD38 (variably decreased), CD45 (dim), CD71 (bright on a major subset), CD117, CD123 (dim), and HLA-DR (dim to absent) without CD34.

AEL, previously known as pure erythroid leukemia in the revised fourth edition WHO Classification, is defined by erythroid predominance comprising ≥80% of bone marrow elements, of which ≥30% are proerythroblasts. AEL is frequently associated with a complex karyotype and biallelic *TP53* alterations [8,117,118]; a subset of abnormal myeloid progenitors may or may not be present. Flow cytometric analysis of AEL has revealed that the neoplastic proerythroblasts express CD36, dim CD45, CD71, and variable CD117, frequently showing decreased to absent CD38, and lacking CD34 expression [46] (Figure 7). Aberrant expression of CD4, CD7, CD13, and HLA-DR is observed in a significant proportion of AEL, features not present in reactive erythroid hyperplasia. CD34-positive myeloblasts in AEL are not increased in number but often exhibit an aberrant immunophenotype, whereas CD34-positive blasts show a normal immunophenotype in conditions associated with reactive erythroid hyperplasia.

#### 3.5.2. Acute Megakaryoblastic Leukemia (AMKL)

**Figure 8:**
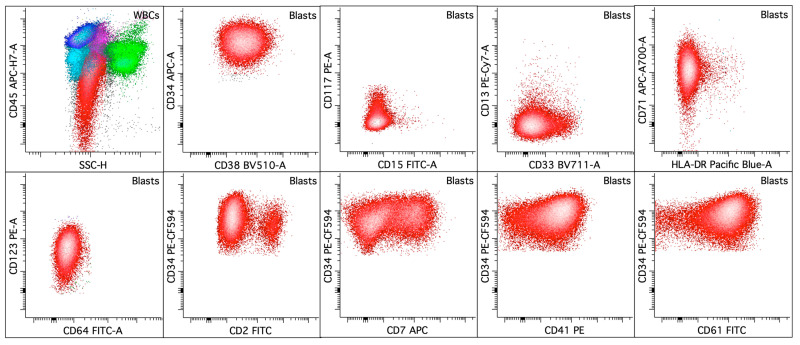
Acute myeloid leukemia with megakaryocytic differentiation. The upper left dot plot displays the total viable white blood cells, with blasts colored in red, lymphocytes in blue, hematogones in aqua, monocytes in pink, and granulocytic cells in green. The rest of the dot plots selectively display leukemic blasts. The leukemic blasts (colored red; ~31% of the white blood cells) in the bone marrow express CD2 (small subset), CD7 (subset), CD33 (dim to mostly absent), CD34 (bright), CD38 (dim), CD41 (dim), CD45 (variably decreased to absent), CD61, CD71, and CD123 (dim) without CD13, CD15, CD64, CD117, or HLA-DR.

AMKL represents 3–5% of AML and is more prevalent in the pediatric population. AMKL arises in children with Down syndrome (DS), children without DS, and adults [8]. The leukemic blasts in AMKL are characterized by megakaryocytic differentiation and express high levels of megakaryocytic markers, including CD42a, CD41, CD61, and/or CD42b, with variable expression of CD4, CD7, CD33, CD34, CD36, CD38, CD56, and CD71 [119] (Figure 8). Expression of CD13, CD117, and HLA-DR is less common in AMKL than in non-AMKL cases. Within AMKL, transient abnormal myelopoiesis (TAM) and myeloid leukemia associated with DS (ML-DS) show higher frequencies of CD34+/CD117+ leukemic blasts compared to other AMKL subgroups. Additionally, ML-DS patients often demonstrate significantly higher expression of CD7, CD11b, CD33, and CD38.

**Figure 9:**
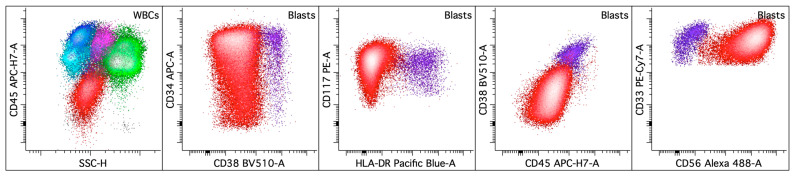
Acute myeloid leukemia with RAM phenotype. The left dot plot displays the total viable white blood cells, with blasts colored in red, lymphocytes in blue, hematogones in aqua, monocytes in pink, and granulocytic cells in green. The rest of the dot plots selectively display leukemic blasts. The leukemic blasts (colored red; ~21% of the white blood cells) in the bone marrow express CD33 (bright), CD34 (variably decreased), CD38 (dim to absent), CD45 (dim to absent), CD56 (bright), and CD117 without HLA-DR. A small normal myeloid blast population (colored purple) is also present.

A specific subset of AML expresses a distinctive immunophenotypic profile referred to as the RAM phenotype, named after the initials of the first patient described with this entity [120]. Patients with the RAM phenotype tend to be younger and have a higher frequency of AMKL, with high induction failure rates and notably poor prognosis. Blasts with a RAM phenotype are characterized by low SSC, bright CD33 and CD56, CD117, dim to absent CD38 and CD45, and absence of CD11b, CD36, and myeloperoxidase [120,121,122] (Figure 9). A consistent feature distinguishing RAM-AML from non-RAM AMKL is the absence of CD36, which is moderately to brightly positive in non-RAM AMKL [121,122]. *CBFA2T3::GLIS2* is the most frequent genetic aberration identified in pediatric non-DS AMKL and associated with an extremely poor outcome [123]. There is a statistical correlation between the RAM phenotype and *CBFA2T3::GLIS2* fusions, indicating a unique genetic driver associated with this phenotype [121,124,125]. Additionally, aberrant cytoplasmic CD3 expression has been described in a subset of pediatric non-DS AMKL with the RAM phenotype and/or *CBFA2T3::GLIS2* fusions [125,126].

The *FOLR1* transcript has been identified as uniquely expressed in AML with *CBFA2T3::GLIS2* fusion, but it is not expressed in most other types of AML and normal CD34+ cells [127]. *FOLR1* encodes folate receptor α, which is overexpressed on the cell surface of blasts. A FOLR1-directed antibody–drug conjugate (ADC) has been developed and showed efficacy against AML with *CBFA2T3::GLIS2* fusion in preclinical studies [128].

#### 3.5.3. Acute Leukemias of Mixed or Ambiguous Lineage (ALAL/MPAL)

**Figure 10:**
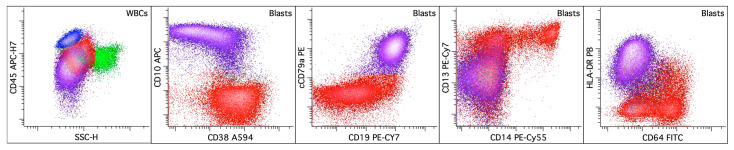
Mixed phenotype acute leukemia, B/myeloid. The left dot plot displays the total viable white blood cells, with two blast populations shown in purple and red, lymphocytes in blue, monocytes in pink, and granulocytic cells in green. The rest of the dot plots selectively display leukemic blasts. The blasts with B lineage differentiation (purple) strongly express B-cell markers CD10, CD19, and CD79a, while lacking monocytic markers CD14 and CD64. In contrast, the blasts with myelomonocytic differentiation (red) variably express CD14 and CD64 and do not express B-cell markers.

In the WHO-HAEM5, ALAL/MPAL is divided into two groups: ALAL with defining genetic abnormalities and ALAL, immunophenotypically defined. Up to 30–40% of ALAL/MPAL cases lack defined genetic abnormalities, making classification exclusively dependent on immunophenotypic criteria. Lineage assignment is optimally achieved using flow cytometry to assess the intensity of the antigen expression relative to normal counterparts at various maturation stages. While the diagnostic criteria for lineage designation of a single blast population remain largely unchanged from the revised fourth edition the WHO Classification, the WHO-HAEM5 immunophenotypic criteria emphasize the intensity and pattern of antigen expression. For lineage confirmation, the intensity of CD19, CD3 (cytoplasmic or surface), and myeloperoxidase on blasts should exceed 50% of the levels seen on normal B-cell progenitors, mature T-cells, and mature neutrophils, respectively [8]. Demonstrating a heterogeneous expression pattern of lineage antigens similar to those on normal progenitors and the coordinated expression of multiple antigens from the same lineage enhances the specificity of the lineage assignment. A definitive cut-off for myeloperoxidase expression by flow cytometry has not been established, with the suggested thresholds ranging from 3% to 28% [129,130,131]. A representative example of B/myeloid MPAL is shown in Figure 10.

## 4. Application of Artificial Intelligence and Machine Learning in Flow Cytometry Data Analysis for AML Diagnosis

Technological advancements have enabled an increased number of fluorochromes/parameters to be measured simultaneously by flow cytometry, leading to greater confidence in defining specific cell populations. However, this increased dimensionality has resulted in complex, labor-intensive data analysis of multiple two-dimensional scatter plots, which is highly dependent on expert knowledge, making reproducible interpretation and standardization challenging. The massive and high-dimensional data, which cannot be adequately handled by manual analysis, have driven the development of novel computational tools to assist in rapid and objective visualization and cell population identification in an automated, standardized process. Flow cytometry data analysis can employ two primary types of computational methods: dimensionality reduction and clustering. Dimensionality reduction techniques allow for the visualization of multidimensional flow cytometry data in two dimensions (2Ds) and a reduction in data size [132,133,134]. Clustering, on the other hand, facilitates the computational detection and quantification of cell populations [135,136,137].

Machine learning (ML) is a branch of artificial intelligence (AI) where algorithms are trained on a given dataset to create a model that can be utilized to analyze and make predictions based on new data [138]. The application of ML models in flow cytometry includes automated diagnosis using automated gating and neural networks for precise classification of cell populations, as well as automated image analysis software powered by AI to assist in the identification and categorization of cells based on their morphological parameters evaluated by flow cytometry. Additionally, automated tools are designed to identify anomalies in flow cytometry data that can confound analysis, such as flowAI, which is implemented as a plugin within flow cytometry analysis software.

In recent years, various modern ML models have been introduced to diagnose and classify acute leukemias, demonstrating promising results in streamlining the diagnosis of AML [22,139,140,141] and other hematolymphoid neoplasms using flow cytometry [142,143,144,145,146]. These models are notable for their ability to significantly decrease the time required for analysis while ensuring statistical correlation with traditional manual diagnostic methods. For instance, an ML model using a graph neural network (GNN) for real-time clinical use showed robust performance in discriminating APL from other AML and achieved 100% accuracy, highlighting the potential for automated data analysis and interpretation to improve diagnostic accuracy and efficiency [19].

Attention-based multi-instance learning models (ABMILMs) are advanced deep learning models designed to perform accurate prediction and classification of samples from individual events [128]. When ABMILMs were applied in flow cytometry data analysis, these models showed strong performance in predicting the presence or absence of acute leukemia, differentiating AML versus acute lymphoblastic leukemia, and the presence or absence of cytogenetic aberrancies and pathologic gene mutations [18]. This study demonstrated the applicability of ML models in the automated diagnosis and classification of AML, providing molecular characterization based on flow cytometry data.

These AI models represent a significant stride forward in automating and refining the accuracy of AML diagnosis, reducing subjectivity, and offering sophisticated decision-making tools. Despite these technical advances and their tremendous potential, clinical implementation of AI solutions remains limited by several key factors. One major restriction is the requirement for large, high-quality, and accurately annotated datasets that capture the diversity and variability of AML cases, which demands expert knowledge. Differences in laboratory practices for data acquisition, such as preanalytical variabilities in sample processing and instrumentation, panel design, and assay validation, can impact the generalizability and reliability of AI models across clinical settings, making it challenging to standardize and integrate data for model training and deployment. The heterogeneity of AML, including the diverse subtypes and immunophenotypic profiles, can lead AI models to overfit on a particular subtype, reducing diagnostic accuracies. Additionally, AI algorithms often operate as a “black-box” and do not provide reasoning for their decision-making processes, which can undermine clinician trust and limit clinical adoption. To support AI as part of a clinical test, the evolving regulatory landscape must provide clear guidelines to ensure that AI applications in clinical flow cytometry maintain high standards of accuracy, reliability, data privacy, and patient safety. The challenges to integrating AI into daily clinical laboratory workflows are varied and often include a lack of computational expertise or resources, uncertainties about practical applications, regulatory frameworks, and the optimal integration of human and AI efforts [147].

## 5. Summary

Flow cytometry is essential for the diagnosis and classification of AML and MRD monitoring post therapy. Strong correlations between immunophenotypic features and underlying genetic aberrations have been recognized, facilitating the prediction of the genotype based on flow cytometry data to guide appropriate workup and prompt clinical decision-making. The application of novel technologies within flow cytometry and the integration of ML models for AML diagnosis aim to enhance diagnostic efficiency and accuracy, reduce reliance on subjective judgment, and assist in informed therapeutic strategies.

## Figures and Tables

**Table 1:** Fifth edition WHO Classification of acute myeloid leukemia.

AML with Defining Genetic Abnormalities
Acute promyelocytic leukemia with *PML::RARA* fusion
AML with *RUNX1::RUNX1T1* fusion
AML with *CBFB::MYH11* fusion
AML with *DEK::NUP214* fusion
AML with *RBM15::MRTFA* fusion
AML with *BCR::ABL1* fusion
AML with *KMT2A* rearrangement
AML with *MECOM* rearrangement
AML with *NUP98* rearrangement
AML with *NPM1* mutation
AML with *CEBPA* mutation
AML, myelodysplasia-related
AML with other defined genetic alterations
**AML, Defined by Differentiation**
AML with minimal differentiation
AML without maturation
AML with maturation
Acute basophilic leukemia
Acute myelomonocytic leukemia
Acute monocytic leukemia
Acute erythroid leukemia
Acute megakaryoblastic leukemia

**Table 2:** Antibody panels used to evaluate acute myeloid leukemia.

Laser	Violet						Blue					Red		
Fluorochrome	PB	SN v428	BV510	BV605	BV711	BV786	A488	FITC	PE	PE-Cy5	PE-Cy7	APC	APC-A700	APC-H7
Myeloid 1	HLA-DR		CD38	CD19	CD33			CD15	CD117		CD13	CD34	CD71	CD45
Myeloid 2	HLA-DR		CD38	CD4		CD14		CD64	CD123		CD13	CD34	CD16	CD45
Myeloid 3	HLA-DR		CD38	CD5			CD56		CD7		CD33	CD34		CD45
Lineage		CD22			cCD3	CD7		cMPO	cCD79a	CD117	CD19	CD34		CD45

APC, Allophycocyanin; BV, Brilliant Violet; FITC, Fluorescein Isothiocyanate; PB, Pacific Blue; PE, Phycoerytherin; PE-Cy5, PE-Cyanine-5; PE-Cy7, PE-cyanine-7.
